# miR-504 Promoted Gastric Cancer Cell Proliferation and Inhibited Cell Apoptosis by Targeting RBM4

**DOI:** 10.1155/2021/5555950

**Published:** 2021-06-04

**Authors:** Yi Zhang, Hongmei Yong, Jing Fu, Guangyi Gao, Huichang Shi, Xueyi Zhou, Mingsheng Fu

**Affiliations:** ^1^Department of Surgical Oncology, Minhang Branch, Fudan University Shanghai Cancer Center, 200240 Shanghai, China; ^2^Department of Oncology, The Affiliated Huai'an Hospital of Xuzhou Medical University and The Second People's Hospital of Huai'an, Huaian 223002, China; ^3^Department of Intensive Care Unit, The Affiliated Huai'an Hospital of Xuzhou Medical University and The Second People's Hospital of Huai'an, Huaian 223002, China; ^4^Department of Gastroenterology, Shanghai Fifth People's Hospital, Fudan University, 200240 Shanghai, China

## Abstract

**Background:**

The purpose of this study was to explore the role and underlying mechanism of miR-504 and RBM4 in gastric cancer.

**Methods:**

The qRT-PCR or Western blot was performed to determine the expressions of miR-504 and RBM4 in the gastric cancer tissues and normal tissues. Human SGC-7901 cells were transfected with miR-504 mimic/inhibitor or pcDNA-RBM4. Cell proliferation and cell apoptosis were assessed by colony formation assay and flow cytometry, respectively. Luciferase reporter gene assays were used to investigate interactions between miR-504 and RBM4 in SGC-7901 cells.

**Results:**

The relative expression of miR-504 was significantly upregulated in the gastric cancer group (*n* = 25) than in the paired normal group (*n* = 25), but the relative RBM4 expression was remarkably downregulated in the gastric tumor group, compared with the normal group. Additionally, miR-504 overexpression increased the viability of gastric cancer cells. Moreover, RBM4 is a functional target of miR-504 in gastric cancer cells. miR-504 was further confirmed to promote SGC-7901 cell proliferation and inhibit cell apoptosis by downregulation RBM4 in vitro.

**Conclusions:**

miR-504 promotes gastric cancer cell proliferation and inhibits cell apoptosis by targeting RBM4, and this provides a potential diagnostic biomarker and treatment for patients with gastric cancer.

## 1. Introduction

Gastric cancer (GC) is the fifth most common malignant neoplasm in humans worldwide and the third leading cause of death among patients with cancer [1]. The early-stage GC patients were treated with various significant therapies. However, the overall prognosis of GC advanced-stage patients remains unfavorable, despite aggressive therapies [2]. Therefore, it is imperative to identify new GC biological markers that will allow us to develop early diagnosis approaches and offer important sights into the targeted treatment for patients with GC.

Recently, the microRNA (miRNA) role on the progression of tumor has been investigated extensively [3]. miRNAs are small noncoding RNA molecules, 19-25 nucleotides in length that regulate mammalian gene expression at the posttranscriptional level [4]. miRNAs can bind to the 3′ untranslated region (UTR) of targeted mRNA, leading to the inhibition of translation and/or the degradation of targeted mRNA [5]. A variety of miRNAs have been shown to regulate the pathogenesis of GC, such as miR-630, miR-532, miR-337-3p, and miR-154 [6–8].

It has been reported that miR-504 plays a tumor suppressive role in certain types of cancer [9–11]. miR-504 can directly bind two sites in the 3′UTR of p53 and negatively regulate human p53 expression [9]. Kikkawa et al. observed that miR-504 inhibited the proliferation of hypopharyngeal squamous cell carcinoma cancer through targeting CDK6 [10]. Yang et al. found that the invasion of oral squamous cell carcinoma cells was regulated by connective tissue growth factor through activating a novel miR-504/FOXP1 signal pathway [11]. However, the possible biological mechanisms of miR-504 in the progression of GC remain unclear.

RNA-binding motif 4 (RBM4), a multifunctional protein, is mainly involved in regulating mRNA translation and alternative splicing [12]. RBM4 plays a vital role in the control of cell apoptosis, proliferation, and migration to inhibit the progression of various tumors, including lung, ovary, and prostate [13]. Yong et al.'s findings suggested that the lower expression of RBM4 was associated with poor overall survival in patients with GC [2]. However, to the best of our knowledge, the molecular mechanisms underlying this association are still not well-documented.

At present, there is no enough data on the potential interaction between miR-504 and RBM4 on GC. In this study, we aim to explore the role of miR-504 and RBM4 in the development of GC, in order to provide significant insights into the diagnosis and treatment of GC.

## 2. Materials and Methods

### 2.1. Clinical Specimens

From July 2019 to July 2020, 25 paired gastric cancer tissues and 25 normal tissues were collected from patients, who received gastric cancer surgery. The lesion tissue was taken during the operation and assigned to the gastric cancer group; the adjacent tissues (distance from the lesion is ≥3 cm) were collected as the control group. Among the patients, 15 were males and 10 were females, aged 45-65 years, with an average age of 55.7 ± 9.8 years, a course of disease of 1-2.5 years, and an average course of 1.8 ± 0.5 years. The stage of tumor lymph node metastasis is the T2N0M01B period. All clinical specimens were reserved at −80°C before the study. The present study was approved by the Affiliated Huai'an Hospital of Xuzhou Medical University and the Second People's Hospital of Huai'an. All patients provided and approved the informed consent.

### 2.2. Cell Culture and Transfection

We obtained human gastric cancer SGC-7901 cells from the Chinese Academy of Sciences (Shanghai, China). SGC-7901 cells were cultured in DMEM at 37°C with 5% carbon dioxide, with 10% FBS (Gibco) and 1% penicillin-streptomycin (Gibco). Lipofectamine 2000 (Invitrogen) was used for cell transfection in accordance with the manufacturer's instructions. We purchased negative control lentiviral vector (LV-NC) and methioninase lentiviral vector (LV-METase) from Shanghai Cancer Institute (Shanghai, China). miR-504 mimic, miR-504 inhibitor, and their negative control were provided by GenePharma Co. Ltd. (Shanghai, China).

### 2.3. RNA Extraction and Quantitative Real-Time PCR

We used RNAiso Plus (Takara, China) to extract total RNA according to the reagent protocol. For the detection of miR-504 and RBM4, PrimeScript™ RT Master Mix (Perfect Real Time) (Takara, China) was utilized to reverse transcribe total RNA into cDNA as the manufacturer's instructions. The qRT-PCR was performed using TB Green® Premix Ex Taq™ (Tli RNaseH Plus) (Takara, China) according to the standard protocol. The relative expression of miR-504 and RBM4 was calculated through the 2^−*ΔΔ*Ct^ method. The internal control for miR-504 and RBM4 mRNA was U6 and GAPDH, respectively. The specific primer sequences obtained from Sangon, China, were listed as follows:

1. miR-504: AGACCCTGGTCTGCACTCTATC
2. U6: GCAAGGATGACACGCAAAT
3. RBM4 (Forward primer): 5′-TGGTCCGGTCATCGAATGTG-3′
4. RBM4 (Reverse primer): 5′-CACACAGCCTTGTGTTCAGC-3′
5. GAPDH (Forward primer): 5′-CCATGGGGAAGGTGAAGGTC-3′
6. GAPDH (Reverse primer): 5′-GAAGGGGTCATTGATGGCAAC-3′

### 2.4. Western Blot Assay

Western blot assay was conducted according to the standard method described in the previous literature [14]. RBM4 (1: 1000) and GAPDH (1: 1000) antibodies were obtained from Cell Signaling Technology (USA).

### 2.5. Double Luciferase Assay

For further gene identification, the double luciferase assay was performed. The full length of the 3′UTR of RBM4 was cloned and amplified. The PCR products were subcloned and inserted in the pmirGLO Dual-Luciferase miRNA Target Expression Vector (Promega Corporation, Madison, Wisc.), named “WT-RBM4” (Wide Type-RBM4). Then, site-directed mutagenesis was performed on the combination site of miR-504 and the target gene gained from bioinformatics software to establish a “MUT-RBM4” (Mutant-RBM4) vector. We utilized Lipofectamine 2000 to cotransfect SGC-7901 cells with the luciferase report vector, which contained the 3′UTR of WT-RBM4 or MUT-RBM4 and miR-504 mimic or miR-504 inhibitor. After SGC-7901 cells were transfected for 24 hours, the luciferase activity was measured as the manufacturer's instructions, using the Luciferase Assay Reporter System (Promega, Madison, WI, USA).

### 2.6. Cell Viability and Apoptosis Analysis

Cell viability was measured by MTT kit (Dojindo, Japan). Briefly, we added 0.5 mg/mL MTT solution to each well for 4 h at different points, after transfection of SGC-7901 cells (5 × 10^3^ cells/well). The medium was removed, and DMSO (150 *μ*L) was added to each well. Then, we measured the absorbance at 490 nm, after 10-mintute oscillation. Additionally, the analysis of cell apoptosis was conducted with the Annexin V-FITC/PI kit (BestBio, Shanghai). In short, after treatment, Annexin V-FITC and PI were used to stain the cells. The cell apoptosis was determined by flow cytometry (BD Biosciences, Franklin Lakes, NJ, USA).

### 2.7. Colony Formation Assay

SGC-7901 cells transfected with LV-anti-miR-504, LV-anti-miR-504+sh-RBM4, or LV-NC were seeded in 6-well plates at a density of 500 cells/well. Then, cells were cultured at 37°C for two weeks. We stained cells with 0.5% crystal violet solution and used PBS to wash them. After cells were photographed, we analyzed the images with ImageJ (NIH) software.

### 2.8. Cycloheximide-Based miR-504 Protein Stability Assay

SGC-7901 cells were transiently transfected for 48 h with LV-NC, LV-anti-miR-504, or LV-anti-miR-504+sh-RBM4. 100 *μ*M of cycloheximide (Sigma-Aldrich, MO) were added to cells, and they were harvested at 0, 1 h, 3 h, and 6 h, respectively. Cells were lysed to produce whole cell lysates, which were subjected to Western blotting.

### 2.9. Statistical Analysis

The data were presented as the mean ± standard deviation. All results were analyzed by SPSS 25.0, using Student's *t*-test or one-way analysis of variance. All experimental analyses were considered statistically significant when *P* < 0.05.

## 3. Results

### 3.1. Relative Expressions of miR-504 and RBM4 in Gastric Cancer Cells

**Figure 1:**
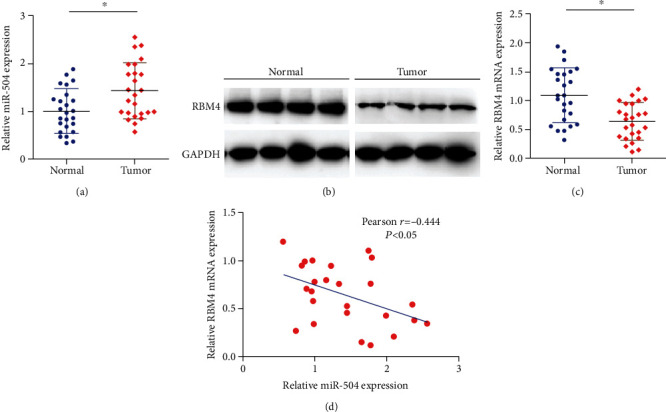
Relative expressions of miR-504 and RBM4 mRNA in normal and gastric cancer tissues. Relative expressions of (a) miR-504 mRNA and (b) RBM4 protein and (c) mRNA in normal (*n* = 25) and gastric cancer tissues (*n* = 25). (d) Pearson correlation analysis of miR-504 and RBM4 expressions in gastric cancer tissues (*n* = 25). ^∗^*P* < 0.05.

The relative expression of miR-504 was significantly upregulated in the gastric cancer group (*n* = 25) than in the paired normal group (*n* = 25) (Figure 1(a)). However, the relative RBM4 mRNA and the protein expression level was remarkably downregulated in the gastric tumor group, compared with the normal group (Figures 1(b) and 1(c)). In addition, the miR-504 expression was negatively correlated with the expression of RBM4 (*r* = −0.444, *P* < 0.05, Figure 1(d)). The dysregulation of miR-504 and RBM4 in gastric cancer cells indicated the important role of miR-504 and RBM4 in the development of gastric cancer.

### 3.2. miR-504 Increased the Viability of Gastric Cancer Cells

**Figure 2:**
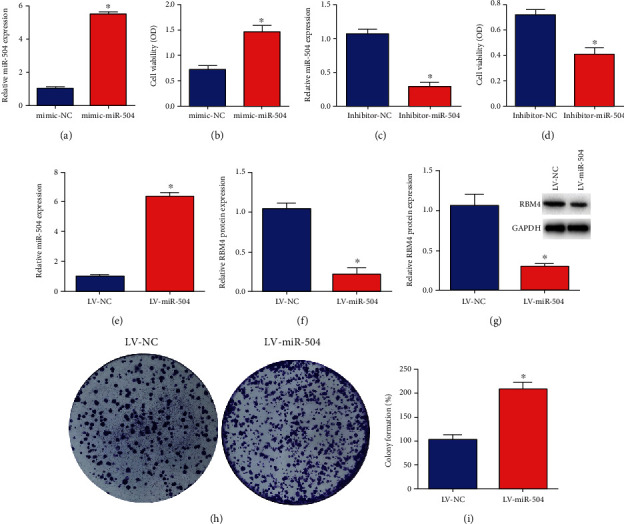
miR-504 increased the viability of gastric cancer cells. (a) Relative expression of miR-504 mRNA in SGC-7901 cells in the mimic-NC group and mimic-miR-504 group. (b) The cell viability of SGC-7901 cells in the mimic-NC group and mimic-miR-504 group. (c) Relative expression of miR-504 mRNA in SGC-7901 cells in the inhibitor-NC group and inhibitor-miR-504 group. (d) The cell viability of SGC-7901 cells in the inhibitor-NC group and inhibitor-miR-504 group. (e) Relative expression of miR-504 mRNA in the LV-NC group and LV-miR-504 group. (f, g) The expression of RBM4 mRNA and protein in the LV-NC group and LV-miR-504 group. (h, i) Colony formation of SGC-7901 cells in the LV-NC group and LV-miR-504 group. ^∗^*P* < 0.05.

To investigate the effect of miR-504 in gastric cancer, we transfected SGC-7901 cells (human gastric cancer cells) with mimic miR-504 or inhibitor miR-504 or the negative controls. The expression of miR-504 and cell viability in gastric cancer cells was remarkably increased via the mimic miR-504 transfection (Figures 2(a) and 2(b)). However, there was a significant reduction in the relative miR-504 expression and cell viability in SGC-7901 cells transfected with the inhibitor miR-504 (Figures 2(c) and 2(d)). The relative miR-504 expression was significantly higher in the LV- miR-504 group than in the LV-NC group, while the level of RBM4 mRNA and protein was evidently lower in the LV-miR-504 group, compared with the LV-NC group (Figures 2(e) and 2(f)). In addition, the rate of colony formation was remarkably increased in the LV-miR-504 group than in the LV-NC group (Figures 2(h) and 2(i)). These results revealed that miR-504 increased the viability of gastric cancer cells.

### 3.3. miR-504 Directly Targeted the RBM4 in Gastric Cancer Cells

**Figure 3:**
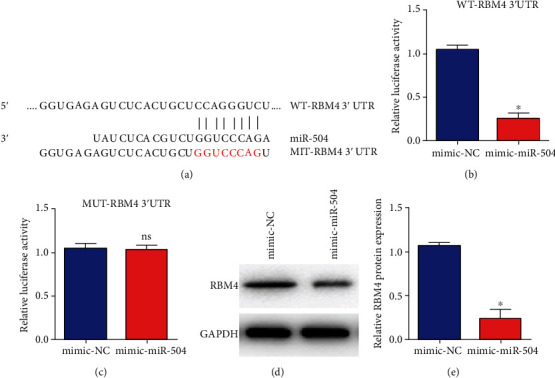
miR-504 directly targeted the RBM4 in gastric cancer cells. (a) Schematic representation of binding sites between miR-504 and RBM4. (b, c) Relative luciferase activity in SGC-7901 cells after transfection with the miR-504 mimic and mimic NC containing the 3′UTR of RBM4 wild type (WT) or mutant type (MUT). (d, e) RBM4 protein expression level in SGC-7901 cells after transfection with the miR-504 mimic or mimic NC. ^∗^*P* < 0.05.

According to the bioinformatics sequence alignment obtained from http://mircoRNA.org/, RBM4 3′UTR had the potential binding sites of miR-504, indicating potential interaction between RBM4 and miR-504 (Figure 3(a)). We constructed and transfected the luciferase reporter plasmids containing the 3′UTR of RBM4 wild type (WT) or mutant type (MUT) into SGC-7901 cells. The results suggested that the expression of relative luciferase activity was remarkably downregulated by miR-504 mimic in SGC-7901 cells with transfection of the reporter gene containing the 3′UTR of WT-RBM4 (Figure 3(b)), while no significant expression changes of miR-504 mimic were observed in terms of the relative luciferase activity in SGC-7901 cells by transfection of the reporter gene containing the 3′UTR of MUT-RBM4 (Figure 3(c)). In addition, the results of western blot analysis showed that overexpression of miR-504 markedly reduced RBM4 protein levels (Figures 3(d) and 3(e)). Therefore, miR-504 directly targeted the RBM4 in gastric cancer cells.

### 3.4. miR-504 Promoted the Growth of SGC-7901 Cells by Downregulation RBM4

**Figure 4:**
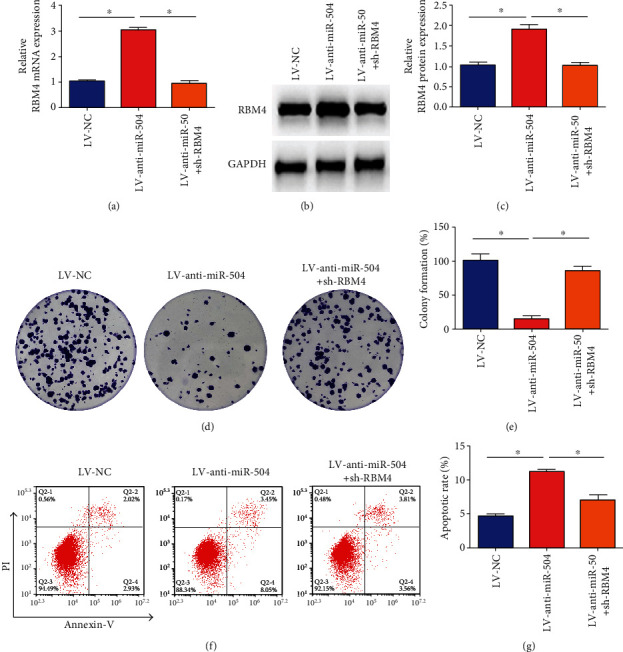
miR-504 promoted the growth of SGC-7901 cells by downregulation RBM4. (a) Relative expression of RBM4 mRNA in each group. (b, c) The expression of RBM4 protein in each group. (d, e) Colony formation of SGC-7901 cells in each group. (f, g) Measurement of the apoptosis rates of SGC-7901 cells by flow cytometry. The Q2-4 and Q2-2 gates were calculated. ^∗^*P* < 0.05.

We transfected SGC-7901 cells with LV-anti-miR-504 only or LV-anti-miR-504 with sh-RBM4 together to explore the miR-504 role on the growth of SGC-7901 cells promoted by RBM4. The level of RBM4 mRNA and protein expression in the LV-anti-miR-504 group was remarkably higher than that in the LV-NC group (Figures 4(a)–4(c)). Additionally, sh-RBM4 significantly reversed the effect of miR-504 inhibitor on the RBM4 protein and mRNA levels in SGC-7901 cells (Figures 4(a)–4(c)). Besides, a significant reduction in the colony formation rate was observed in the LV-anti-miR-504 group, compared with the LV-NC group. However, the rate of colony formation in the LV-anti-miR-504+sh-RBM4 group was relatively higher than that of the LV-anti-miR-504 group (Figures 4(d) and 4(e)). FACS analysis indicated that the combined early and late apoptotic cell rate was significantly increased in the LV-anti-miR-504 group than in the control group, while the apoptotic rate of SGC-7901 cells in the LV-anti-miR-504+sh-RBM4 group was markedly lower than that in the LV-anti-miR-504 group (Figures 4(f) and 4(g)). These results verified that miR-504 promoted GC cell proliferation and inhibited cell apoptosis by downregulation of RBM4.

### 3.5. miR-504 Suppressed SGC-7901 Cell Apoptosis via Targeting RBM4

**Figure 5:**
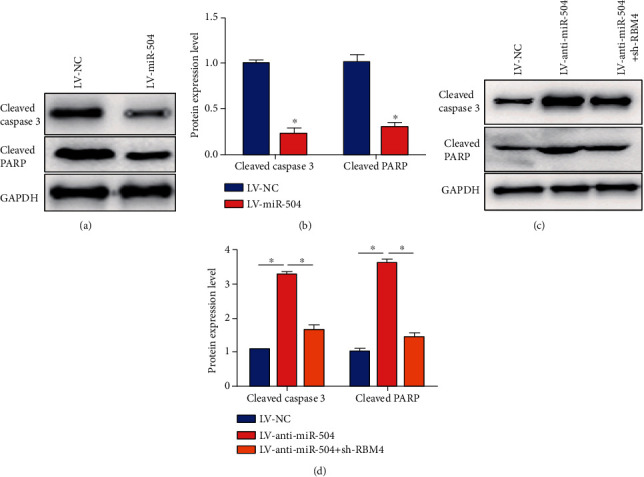
miR-504 suppressed SGC-7901 cell apoptosis via targeting RBM4. (a, b) Relative protein expression of cleaved caspase 3 and cleaved PARP in the LV-NC group and LV-miR-504 group. (c, d) Relative protein expression of cleaved caspase 3 and cleaved PARP in each group. ^∗^*P* < 0.05.

The proapoptosis mediators and molecular markers were examined to explain the molecular basis of SGC-7901 cell apoptosis. The protein expression level of the cleaved caspase 3 and cleaved PARP was significantly lower in the LV-miR-504 group, compared with the LV-NC group (Figures 5(a) and 5(b)). The results of western blot assay showed that LV-anti-miR-504 remarkably increased the protein expression level of the cleaved caspase 3 and cleaved PARP in the LV-anti-miR-504 group, compared with the LV-NC group (Figures 5(c) and 5(d)). Nevertheless, the cleaved caspase 3 and cleaved PARP protein level in the LV-anti-miR-504+sh-RBM4 were lower than those in the LV-anti-miR-504 group (Figures 5(c) and 5(d)). These data revealed that miR-504 suppressed SGC-7901 cell apoptosis via targeting RBM4.

## 4. Discussion

In the present study, we found that the expression of miR-504 was upregulated in GC cells, and it promoted GC cell proliferation and inhibited cell apoptosis by targeting RBM4. Our findings suggested that miR-504 functioned as a tumor promoter gene in the progression of GC. miR-504 has been reported to demonstrate significant associations in the development of many human cancers [15]. Gao et al. observed that miR-504 promoted the proliferation and metastasis of breast cancer cell via targeting BRMS1 [16]. In addition, mir-504 promoted the growth and metastasis of human osteosarcoma via targeting TP53INP1 [17]. To our knowledge, this was the first study to investigate the functional role of miR-504 and RBM4 in GC.

Further, this study revealed that miR-504 directly targeted the RBM4 in GC cells. RBM4 can regulate alternative splicing and exon selection in splicing models [2]. RBM4 inhibited cell proliferation as well as migration in a variety of cancers through the specific control of cancer-related splicing [18]. Previous studies showed that RBM4 was reduced in GC cells and it suppressed the growth and metastasis of GC tumor [19]. Moreover, it was reported that lower RBM4 expression had a close relationship with reduced overall survival and disease-free survival in GC patients [2]. Our study showed that RBM4 was a new target of miR-504. Besides, RBM4 was negatively mediated via miR-504 in GC cells. The present study revealed that miR-504 inhibited GC cell growth by downregulation of RBM4. Future studies are needed to elucidate the potential underlying mechanisms.

Our findings suggested a novel, potential mechanism of miR-504 in GC. In addition, we observed that miR-504 suppressed GC cell survival via mediating RBM4. Further understanding of the miR-504 underlying mechanism in GC will make a significant contribution to the development of effective treatment for GC, and this study was expected to provide an experimental foundation for targeted therapies of GC.

## 5. Conclusions

In summary, miR-504 promotes GC cell proliferation and inhibits cell apoptosis by targeting RBM4, and this provides a potential diagnostic biomarker and treatment for patients with GC.

## Data Availability

All materials and data of this study are available and can be requested from the corresponding author.
